# Earning Power in Women Workers: A Momentous Opportunity

**Published:** 1916-12-16

**Authors:** 


### THE MATRONS' AND SISTERS' DEPARTMENT.

### EARNING POWER IN WOMEN WORKERS.

### A Momentous Opportunity.

A lifelong familiarity with hospitals, and the men and women who devote much time to service of all kinds within their walls, has made us wonder how many of them, as individuals, have really given a thought to the earning power with which they were endowed at birth. The case of the men must be left for consideration in another section of The Hospital on a future occasion. The women workers include probably every type of womankind in the Empire. It would be a great asset for the race, if we could have a book, in which were recorded the occasion, the circumstances, the real, actual reason how, when, and why, each woman worker first became connected with a hospital.

Thirty years ago, weighing those who held responsible positions or who were engaged in responsible nursing work, it could have been said with truth that the majority of them had from their birth the instinct of the true Samaritan, and that attendance on the sick, where they could realise by faithful service, that they were actually rendering appreciable aid to the suffering and the helpless, yielded an inspiration and satisfaction which no other opening for women presented. At that time, for this very reason, though the women workers engaged in hospitals compared with those of to-day were relatively few, they included a number possessing the talents of administration, sterling common-sense, self-control, the power to dispense sympathy in the best meaning of this much-abused word, and the will to work early and late, in season and out of season, if only some poor sufferer, by their ministrations, could be freer from pain or helped to recover. To this type belonged Agnes Jones, Alice Fisher, Katherine Monk, and others who stand out still and will be ever memorable as types of great women with noble souls and splendid powers for fruitful work.

Thirty years have changed many things, amongst others the type and numbers of workers for the sick, who now represent a great army. No doubt the old spirit is present still, but there are only a certain number of people of either sex born each year with the high talents in question, and the great multiplication of opportunities for women workers, in an ever-increasing number of fields of labour, must tend to diminish rather than to increase the total of those with the talents, who at present enter for the nursing profession. And the modern nursing army, with its cheery, enthusiastic workers, have amongst the possessors of its best brains, a material number of those who have failed to appreciate the earning powers they possess. Where this appreciation is absent, there results a failure to cultivate and use this special font of grey matter, and to keep it well under control so that it may be held in reserve or put forward with energy, as circumstances may offer. We believe, as a matter of observation and study of our fellow men and women, that the reserve earning power unutilised by men and women to-day in Great Britain represents a cash value of many million pounds sterling per annum.

These reflections had their origin at a recent consultation where it was essential to determine, how a woman of intelligence could most readily sell her work for a sum equal to her needs. The points submitted for our consideration as a referee included most occupations open to women, but not a word or thought of the actual earning power of the individual worker whose case was presented. Yet, to-day, the way to live and to thrive in this country is, to take care to arrive at a just appreciation of our own earning powers. What the Tribunals do for the Army by bringing knowledge and thought to bear on the men of the nation qualified to engage in war, should be done for others of both sexes by a school of economics, which should possess a Professor of Earning Power, so that every man and woman might have an opportunity to study and develop the gift of appreciating its value.

Women workers in hospitals do not appreciate this power, and so cannot at present use their earning powers effectively. They have not an idea of some gifts they possess, and so are apt to settle down into some department or section and there remain. Others with apprehension make it their first business to learn the work of every department, though the earning-power idea may be unknown to them. These latter are entitled to feel that they have educated themselves to a point of high efficiency, and it should be easy for them to study earning power and benefit by it.

There is some nurse, in every hospital, institution, co-operation, club, and establishment where nurses are associated, who, for instance, possesses the talent to take up the *Nursing Mirror* scheme for awakening the nurses to defend their special rights and win victory for State Recognition and Registration by joining the College of Nursing, now. Why should not each matron and lady superintendent, and each ambitious woman worker in these institutions, by way of experiment, study earning power to the extent of finding, or encouraging, or aiding every worker with the gift of leadership, to secure speedy victory for the College of Nursing? Earning power can at least claim to be a novel idea, well worthy of cultivation. Christmas and the New Year, with the extra work which both entail, might prove an excellent season for the experiment here suggested, for it can afford real relaxation with a maximum of interest. Is there a woman worker in any institution who does not wish to cultivate her intelligence, and so to gain mental power in the direction of increased grey matter and diminished fibrous tissue?

